# Expression and regulation of long noncoding RNAs during the osteogenic differentiation of periodontal ligament stem cells in the inflammatory microenvironment

**DOI:** 10.1038/s41598-017-14451-4

**Published:** 2017-10-25

**Authors:** Qingbin Zhang, Li Chen, Shiman Cui, Yan Li, Qi Zhao, Wei Cao, Shixiang Lai, Sanjun Yin, Zhixiang Zuo, Jian Ren

**Affiliations:** 1 Key Laboratory of Oral Medicine, Guangzhou Institute of Oral Disease, Stomatology Hospital of Guangzhou Medical University, Guangzhou, 510140 China; 2 Sun Yat-sen University Cancer Center, State Key Laboratory of Oncology in South China, Collaborative Innovation Center for Cancer Medicine, Sun Yat-sen University, Guangzhou, 510060 China; 3 State Key Laboratory of Biocontrol, School of Life Sciences, Sun Yat-sen University, Guangzhou, 510275 China; 4 Health Time Gene Institute, Shenzhen, 518000 China

## Abstract

Although long noncoding RNAs (lncRNAs) have been emerging as critical regulators in various tissues and biological processes, little is known about their expression and regulation during the osteogenic differentiation of periodontal ligament stem cells (PDLSCs) in inflammatory microenvironment. In this study, we have identified 63 lncRNAs that are not annotated in previous database. These novel lncRNAs were not randomly located in the genome but preferentially located near protein-coding genes related to particular functions and diseases, such as stem cell maintenance and differentiation, development disorders and inflammatory diseases. Moreover, we have identified 650 differentially expressed lncRNAs among different subsets of PDLSCs. Pathway enrichment analysis for neighboring protein-coding genes of these differentially expressed lncRNAs revealed stem cell differentiation related functions. Many of these differentially expressed lncRNAs function as competing endogenous RNAs that regulate protein-coding transcripts through competing shared miRNAs.

## Introduction

Periodontal diseases, such as periodontitis, are the most common causes of tooth loss^1^. Periodontal regeneration via the application of stem cells is the future direction of therapy for periodontal diseases. To date, several types of stem cells, including mesenchymal stem cells (MSCs), embryonic stem cells (ESCs) and induced pluripotent stem cells (iPSCs), have been investigated for periodontal regeneration. MSCs are gaining more appreciation for their application in periodontal regeneration because they are not subject to ethical issues^2–5^. MSCs have the potential to be involved in multiple types of differentiation, including neurogenic, cardiomyogenic, chondrogenic and osteogenic differentiation. Periodontal ligament stem cells (PDLSCs) are a type of MSC. PDLSCs are more suited for maintenance and regeneration of periodontal tissues, compared to other mesenchymal stem cells, such as bone marrow stromal cells (BMSCs)^3,6^. Several factors have been shown to regulate the potency of PDLSCs, including tissue origin^7^, age of the donor^8^, inflammatory condition^9^ and growth factors^10^. Increasing evidence shows that inflammation hampers the osteogenic differentiation potency of PDLSCs^9,11^. Therefore, determining the molecular mechanism of osteogenic differentiation of PDLSCs in the inflammatory microenvironment is critical for tooth regeneration.

In recent years, long noncoding RNAs (lncRNAs) have attracted much attention. Due to recent advances in transcriptome sequencing, it has been realized that a large portion of the genome can be transcribed^12^. Tens of thousands of lncRNAs have been identified in mammalian genomes during diverse biological processes from development to immunity^13–15^. Although initially recognized for the roles of dosage compensation and genomic imprinting, lncRNAs are increasingly implicated in a wide range of human diseases, such as autoimmune disease and cancer.

Over the last few years, several investigations of the functions of lncRNAs in stem cells indicated that lncRNAs play critical roles in the differentiation of stem cell populations. Loewer. *et al*. uncovered 133 and 104 lncRNAs that were significantly increased and repressed, respectively, in iPSCs and ESCs compared with fibroblasts^16^. They further demonstrated that one such lncRNA (lncRNA-RoR) could modulate stem cell reprogramming. Ng *et al*. carried out an lncRNA microarray expression profiling to find differentially expressed lncRNAs in human ESC and human neural progenitor cells (NPC), which resulted in 36 lncRNAs that showed significant overexpression in hESCs compared to NPCs^17^. Several studies have investigated the functions of lncRNAs in MSC-derived lineages. Zhuang *et al*. revealed that upregulation of the lncRNA MEG3 promotes osteogenic differentiation of MSCs from multiple myeloma^18^. Dong *et al*. found 457 and 513 lncRNAs that were significantly upregulated or downregulated, respectively, in PDLSCs compared to BMSCs^19^. Jia *et al*. demonstrated that the lncRNA ANCR was a key regulator of the proliferation and osteogenic differentiation of PDLSCs^20^. To date, little is known regarding the functions of lncRNAs during the osteogenic differentiation of PDLSCs in the inflammatory microenvironment, and the systematic characterization of lncRNAs is lacking.

To address this, we systematically explored the expression and regulation of lncRNAs during the osteogenic differentiation of PDLSCs in the inflammatory microenvironment by high-throughput RNA sequencing (RNA-seq) experiments. We first evaluated the effect of the inflammatory factor TNF-α on the osteogenic differentiation of PDLSCs. We then performed RNA-seq in three subsets of PDLSCs composed of undifferentiated PDLSCs (uPDLSCs), differentiated PDLSCs without TNF-α stimulation (dPDLSCs) and differentiated PDLSCs under TNF-α stimulation (TNF-α-dPDLSCs). We systematically characterized PDLSC-related lncRNAs. By using qRT-PCR, we validated the expression pattern of some lncRNAs. Overall, we provided a valuable resource of lncRNA expression profiling in PDLSCs and highlight the importance of lncRNAs during osteogenic differentiation of PDLSCs in the inflammatory microenvironment.

## Results

### Systematical identification of lncRNAs in PDLSCs

**Figure 1:**
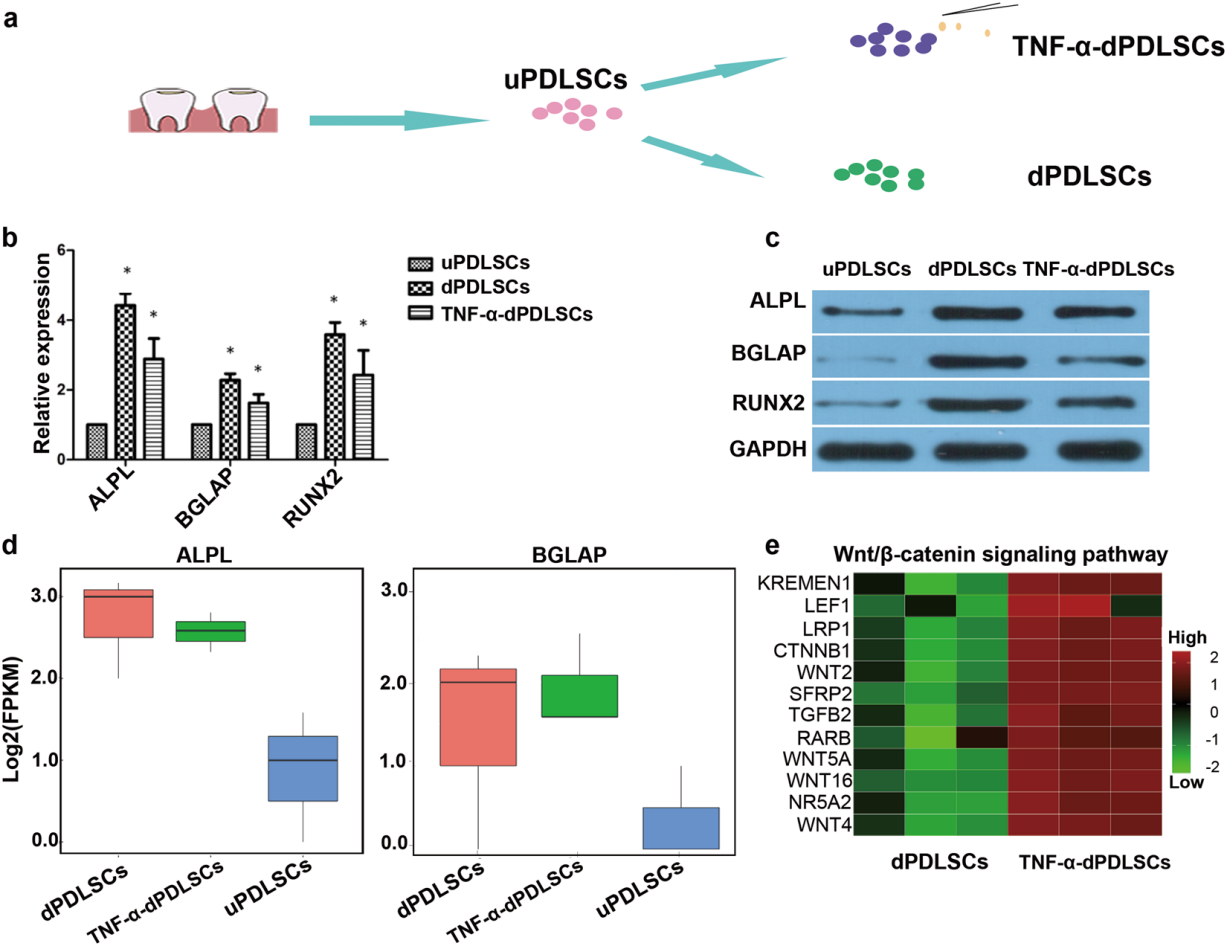
The osteogenic differentiation was suppressed by an inflammatory microenvironment. (**a**) The PDLSC subsets used for RNA sequencing analysis. The cells were isolated from the middle part of the root surface from premolar teeth and cultured for approximately 3-4 generations until the cells exhibited stem cell characteristics (uPDLSCs). With or without induction of TNF-α for 7 days, uPDLSCs were subcultured into dPDLSCs and TNF-α-dPDLSCs. (**b**) The expression of the osteogenic differentiation markers ALPL, BGLAP and RUNX2 in uPDLSCs, dPDLSCs and TNF-α-dPDLSCs was determined by quantitative RT-PCR. (**c**) The expression of the osteogenic differentiation markers ALPL, BGLAP and RUNX2 in uPDLSCs, dPDLSCs and TNF-α-dPDLSCs was determined by western blot. (**d**) The expression of the osteogenic differentiation markers ALPL and BGLAP was determined by RNA-Seq. (**e**) Heatmap showing the expression of genes involved in the Wnt/β-catenin signaling pathway that were significantly upregulated in TNF-α-dPDLSCs compared to dPDLSCs.

We performed polyA selected RNA sequencing (RNA-Seq) using three distinct PDLSC cell types composed of undifferentiated PDLSCs (uPDLSCs), differentiated PDLSCs without TNF-α stimulation (dPDLSCs) and differentiated PDLSCs under TNF-α stimulation (TNF-α-dPDLSCs). Firstly, we isolated the cells from the middle part of the root surface of the premolar teeth. After approximately 3–4 generations of culture, the cells exhibited typical PDLSC characteristics, such as a spindle-shaped morphology (Supplementary Fig. 1A). Then, PDLSCs were subcultured with or without induction of TNF-α (Fig. 1a). After 21 days in culture, we detected that dPDLSCs were more differentiated than TNF-α-dPDLSCs, which was characterized by number, size and color of mineralized nodules (Supplementary Fig. 1B and C). The osteogenic differentiation markers *RUNX2*, *BGLAP* and *ALPL* were also significantly downregulated in the TNF-α-dPDLSC group compared to the dPDLSC group at both the mRNA and protein level (Fig. 1b, Fig. 1c). Taken together, the inflammatory microenvironment could suppress the osteogenic differentiation of PDLSCs as demonstrated by previous studies. We harvested the three subsets of PDLSCs, with three biological replicates in each subset, for transcriptome sequencing. According to the transcriptome sequencing result, the expression patterns of protein-coding genes and pathways known to have functional roles in osteogenic differentiation were consistent with previous studies. The expression of the known osteogenic differentiation markers *ALPL* and *BGLAP* was lowest in uPDLSCs and highest in dPDLSCs (Fig. 1d). Moreover, we observed that most of the genes in the Wnt pathway were significantly upregulated in the TNF-α-dPDLSCs compared to the dPDLSCs (Fig. 1e), which is consistent with previous studies^11^. The above results confirm the validity of our cell culture and sequencing experiments.

**Figure 2:**
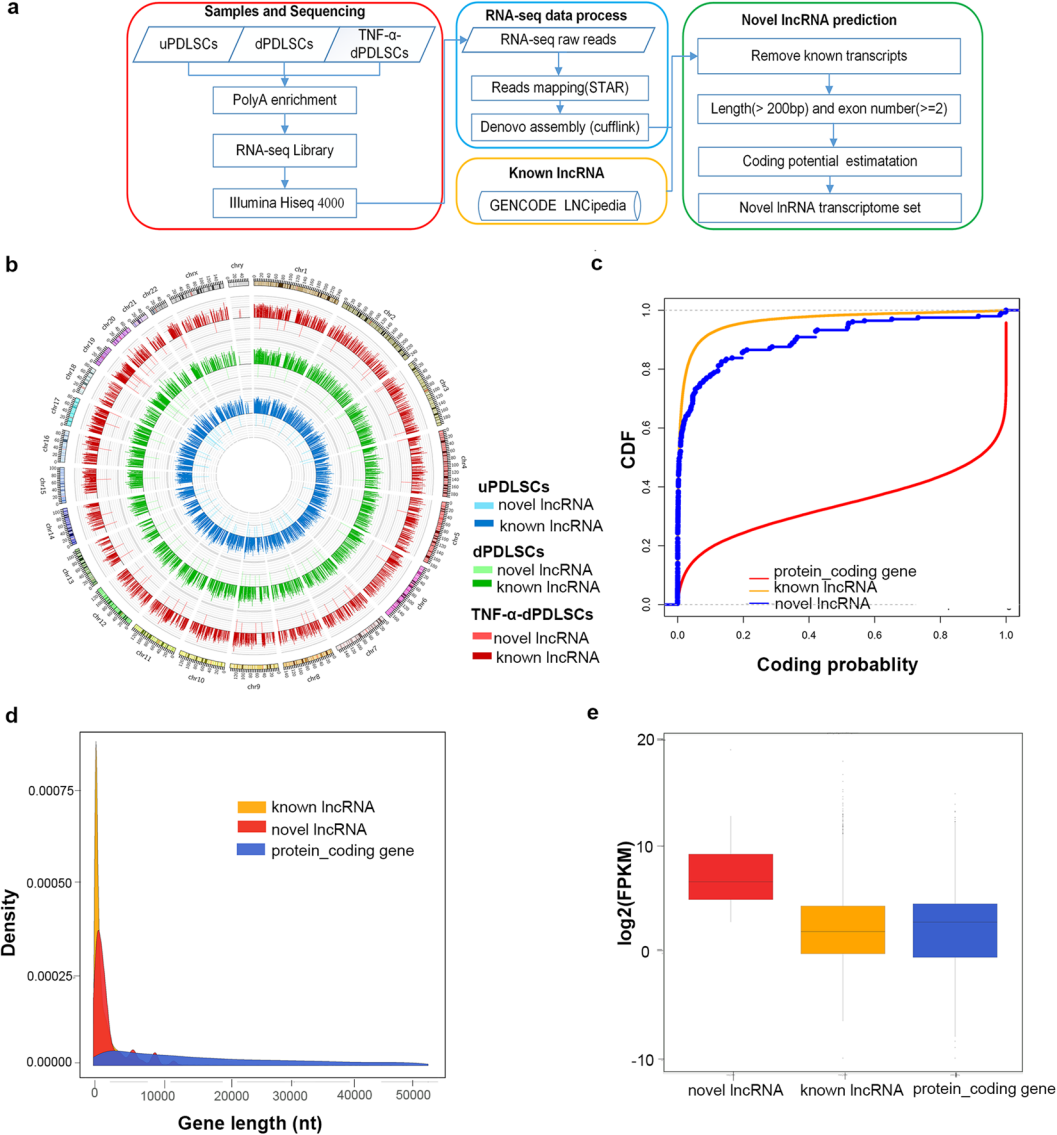
Systematic identification of lncRNAs in PDLSCs. (**a**) The pipeline for the identification of novel lncRNAs. (**b**) Circos plot showing the lncRNA profiling in PDLSCs. Each circle represents a PDLSC subset (from inner to outer: uPDLSCs, dPDLSCs and TNF-α-dPDLSCs). For each circle, the inside bars represent novel lncRNAs and the outside bars represent known lncRNAs. (**c**) Coding potential of transcripts from the entire dataset, estimated using CPAT algorithm. (**d**) Length distribution of protein-coding genes, known lncRNAs and novel lncRNAs. (**e**) Expression abundance of protein-coding genes, known lncRNAs and novel lncRNAs.

We then identified lncRNAs using a strategy similar to one previously published^14^ (Fig. 2a). After de novo transcriptome assembly of the RNA-seq reads, we removed the transcripts that had sense exonic overlap with known protein-coding genes and only kept the long (>200 bp) transcripts with no less than two exons. We then analyzed the protein-coding potential of the remaining transcripts using two computational tools: PLEK^21^ and CPAT^22^. The transcripts that were not predicted as protein coding by either of the two methods were retained as candidate lncRNAs. The candidate lncRNAs were merged with previous lncRNAs from Gencode^12^ and Lncipedia^23^ to build a final lncRNA reference. This final lncRNA reference consisted of a total of 31,885 lncRNAs, which included 63 novel lncRNAs not annotated in the existing lncRNA databases (Fig. 2b, Supplementary Table 1).

Coding potentials for novel lncRNAs, annotated lncRNAs and protein-coding genes were assessed using CPAT for comparison. As a result, novel lncRNAs and annotated lncRNAs showed low average coding potential scores in comparison with higher average coding potential scores characterizing the protein-coding genes (Fig. 2c). The length density distribution of the identified novel lncRNAs was similar to that of annotated lncRNAs, and lncRNAs are on average shorter than protein-coding genes (Fig. 2d). Of note, the novel lncRNAs had more abundant expression in these PDLSC populations than that of annotated lncRNAs and protein-coding genes (Fig. 2e).

### Integrative analysis reveals potential functions of novel lncRNAs in PDLSCs

**Figure 3:**
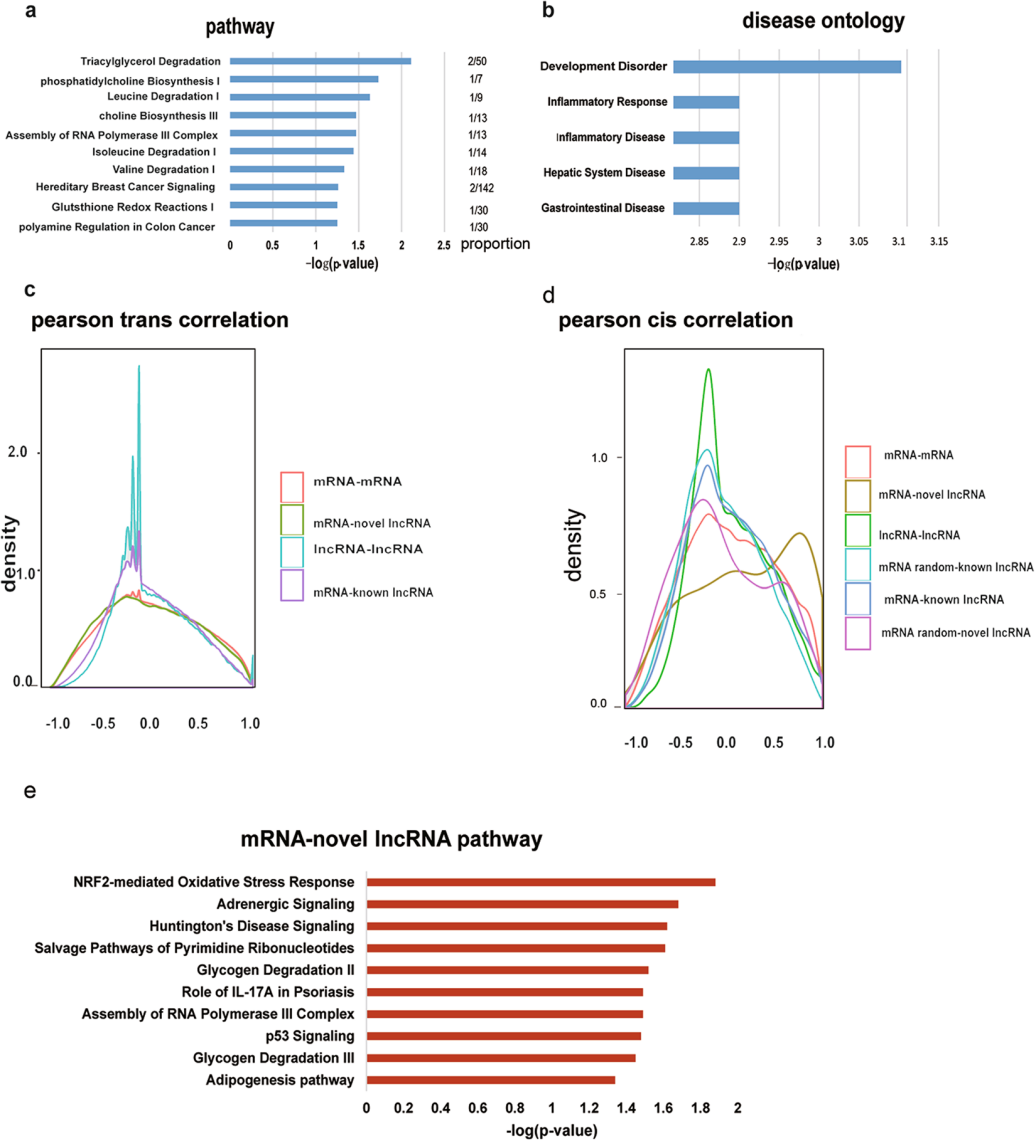
Annotation of novel lncRNAs. (**a**) The top ten significantly enriched KEGG pathways for protein-coding genes near novel lncRNAs. (**b**) The top five disease ontologies of protein-coding genes near novel lncRNAs. (**c**) Density histograms of pairwise Pearson expression correlations between genes of different classes in trans. (**d**) Density histograms of pairwise Pearson expression correlations between genes of different classes in cis. (**e**) The top ten significantly enriched pathways for mRNAs cis positively correlated with novel lncRNAs (Pearson correlation > 0.7).

To uncover the potential functions of the novel lncRNAs in PDLSCs, we systematically examined the function of protein-coding genes located near the novel lncRNAs. IPA analysis revealed that the functions of protein-coding genes near novel lncRNAs were significantly enriched in amino acid metabolism pathways, such as leucine degradation, isoleucine degradation and valine degradation (Fig. 3a). Nutritional effects, particularly amino acid metabolism, are known to play important roles in the maintenance and differentiation of mesenchymal stem cells^24^. Our results suggested that the lncRNAs newly identified in PDLSCs are largely involved in regulating amino acid metabolism during osteogenic differentiation. Furthermore, IPA analysis also revealed that the neighboring protein-coding genes were significantly related to development disorders and inflammatory diseases (Fig. 3b). The above results demonstrated that novel lncRNAs are not randomly located in the genome but are preferentially located near genes with particular functions, suggesting that these novel lncRNAs play key regulation roles in PDLSCs.

Correlation analysis is widely used to infer the function of lncRNAs. To further explore the potential function of novel lncRNAs, we systematically investigated co-expression patterns of lncRNAs and protein-coding genes. To do this, we computed pairwise expression correlations between genes across all samples. Expression correlations were composed of trans correlations (defined as gene pairs separated by 1 > MB distance or located on different chromosomes) and cis correlations (defined as gene pairs located within a 100-kB genomic region). The trans pairs tend to be negatively correlated. Of 147,925,919 trans correlation pairs tested, 12.33% of the pairs had a Pearson correlation coefficient >0.5, versus 44.82% of pairs with Pearson correlation coefficients < −0.5 (Fig. 3c). For cis pairs, we found that novel lncRNAs and protein-coding genes were mostly positively correlated, indicating the functional importance of these novel lncRNAs in PDLSCs (Fig. 3d). Gene set enrichment analysis indicated that protein-coding genes that were strongly positively correlated with novel lncRNAs were mainly enriched in the *NRF2*-mediated oxidative stress response pathway (Fig. 3e). Nuclear factor erythroid 2-related factor 2 (*NRF2*), as a well-established global regulator of the oxidative stress response, plays a regulatory role in the maintenance of stem cell functions^25^. The novel lncRNAs that are highly correlated with the *NRF2* pathway may participate in the *NRF2*-mediated oxidative stress response during differentiation of PDLSCs.

### Differential expression of lncRNAs during osteogenic differentiation

**Figure 4:**
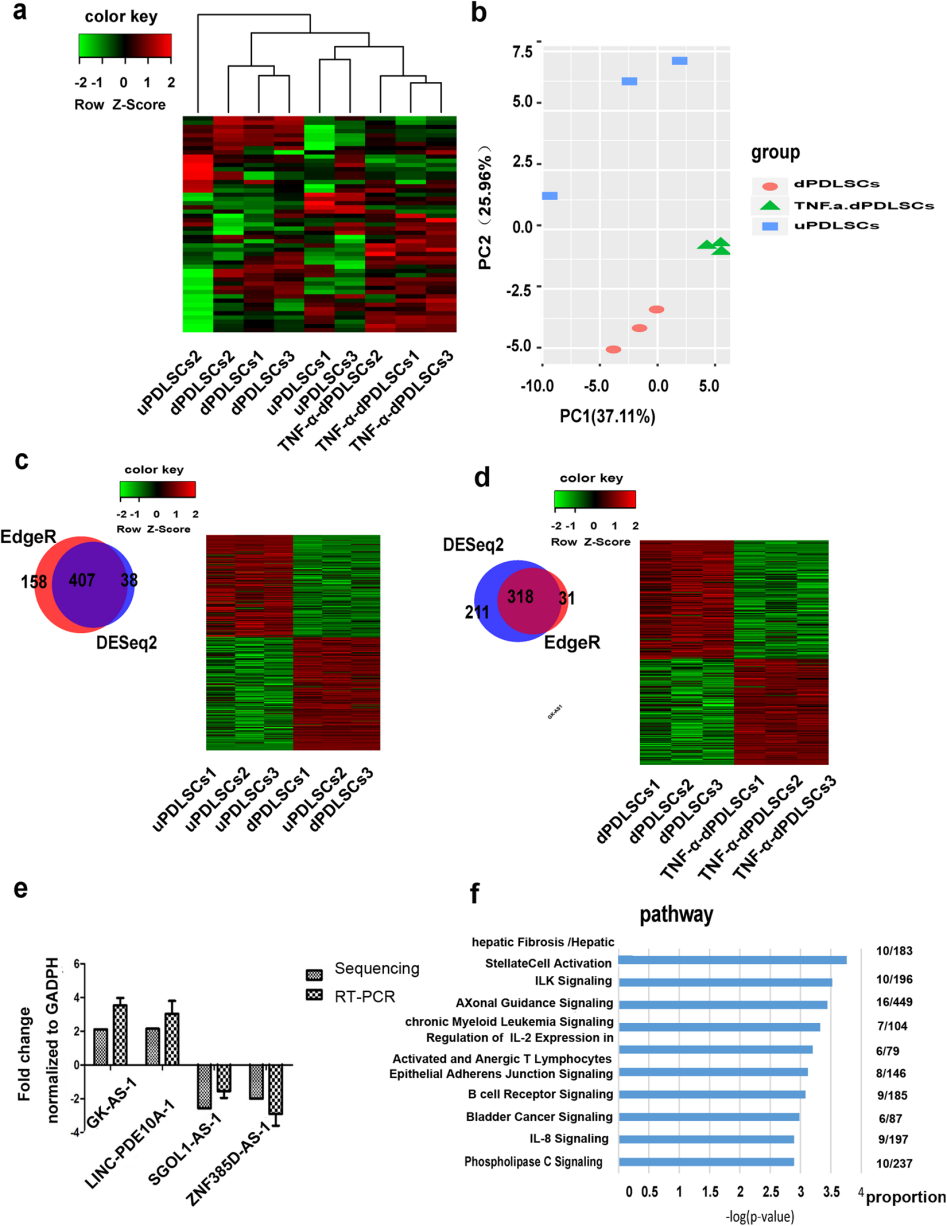
Differential expression of lncRNAs during osteogenic differentiation. (**a**) Heatmap showing the hierarchical clustering result of all expressed lncRNAs of the three PDLSCs subsets. (**b**) The principal component analysis of three cell types based on all expressed lncRNAs of the three subset types. (**c**) The number of differentially expressed (DE) lncRNAs between uPDLSCs and dPDLSCs detected by EdgeR and DESeq2 are shown in the Venn plot. Heatmap showing the overlapping DE lncRNAs between EdgeR and DESeq2. (**d**) The number of differentially expressed (DE) lncRNAs between dPDLSCs and TNF-α-dPDLSCs detected by EdgeR and DESeq2 are shown in a Venn plot. Heatmap showing the overlapping DE lncRNAs between EdgeR and DESeq2. (**e**) The fold changes of four lncRNAs (*CK-AS1, LINC-PDE10A-1, SGOL1-AS-1, ZNF3650-AS-1*) measured by qRT-PCR and sequencing. (**f**) Top ten KEGG pathways for neighboring protein-coding genes for DE lncRNAs between TNF-α-dPDLSCs and dPDLSCs.

We then determined the lncRNAs related to osteogenic differentiation of PDLSCs in an inflammatory microenvironment. LncRNAs are considered to be tissue/time-course specific. In this regard, we looked at the novel lncRNAs first. We first performed hierarchical clustering of the PDLSC samples based on the expression of the novel lncRNAs. We observed that dPDLSC and TNF-α-dPDLSC samples were separated into distinct clusters by hierarchical clustering (Fig. 4a). The principal component analysis revealed three distinct clusters, with each cluster representing a cell type (Fig. 4b). These results demonstrated that the novel lncRNAs were dynamically regulated during osteogenic differentiation, and the expression of a large number of novel lncRNAs was changed upon TNF-α stimulation.

To specifically identify those lncRNAs related to osteogenic differentiation, we then performed differential expression analysis among uPDLSCs, dPDLSCs and TNF-α-dPDLSCs using DESeq. 2 and EdgeR. Only the overlapped, differentially expressed lncRNAs identified from DESeq2 and EdgeR were retained. We identified 214 upregulated lncRNAs and 193 downregulated lncRNAs in dPDLSCs compared to uPDLSCs (Fig. 4c, Supplementary Table 2), and we identified 149 upregulated lncRNAs and 169 downregulated lncRNAs in TNF-α-dPDLSCs compared to dPDLSCs (Fig. 4d, Supplementary Table 3). Overall, 650 lncRNAs were differentially expressed among the three PDLSC subpopulations. Using RT-PCR, we validated two upregulated lncRNAs and two downregulated lncRNAs in TNF-α-dPDLSCs compared to dPDLSCs. The fold changes of the two upregulated lncRNAs (LINC-PDE10A-1 and GK-AS-1) and the two downregulated lncRNAs (ZNF385D-AS-1 and SGOL1-AS-1) were in accordance with that of the RNA-seq results (Fig. 4e). At the same time, we also identified the differentially expressed protein-coding genes (742 differentially expressed protein-coding genes between uPDLSCs and dPDLSCs, Supplementary Table 4; 354 differentially expressed protein-coding genes between dPDLSCs and TNF-α-dPDLSCs, Supplementary Table 5). We validated the expression change of four protein-coding genes using RT-PCR (Supplementary Fig. 2).

We then performed pathway enrichment analysis for the neighboring protein-coding genes of these differentially expressed genes using IPA. We found that the neighboring protein-coding genes for DE lncRNAs between uPDLSCs and dPDLSCs were mainly distributed in the dTMP de novo biosynthesis pathways and serotonin receptor signaling (Supplementary Fig. 3). Previous studies have reported that serotonin receptors were necessary for the recruitment of pulpal cells involved in dental repair^26^. The mechanism and function of the serotonin receptor signaling-related DE lncRNAs including *GCH1-AS, HTR1B-AS, LINC-ADCY1* and *ADCY3-AS* needs further study. The neighboring protein-coding genes for the DE lncRNAs between TNF-α-dPDLSCs and dPDLSCs were mainly in the inflammatory process such as IL-2 signaling and IL-8 signaling (Fig. 4f). Apart from the pathway enrichment analysis for neighbouring protein-coding genes of the differentially expressed lncRNAs using IPA, we have further performed LncRNAs2Pathway analysis for these differentially expressed lncRNAs. Pathways enriched for DE lncRNAs between uPDLSCs and dPDLSCs were listed in Supplementary Table 6A. Pathways enriched for DE lncRNAs between dPDLSCs and TNF-α-dPDLSCs were listed in Supplementary Table 6B. Interestingly, the pathways enriched in DE lncRNAs between uPDLSCs and dPDLSCs included glycosaminoglycan and glycosphingolipid biosynthesis which were known to be related to osteogenic differentiation (Supplementary Fig. 4). Oxidative phosphorylation, which is enriched in DE lncRNAs between dPDLSCs and TNF-α-dPDLSCs, has been reported to play significant roles both in osteogenic differentiation and inflammation (Supplementary Fig. 4).

### lncRNA-mRNA related ceRNA network during osteogenic differentiation

**Figure 5:**
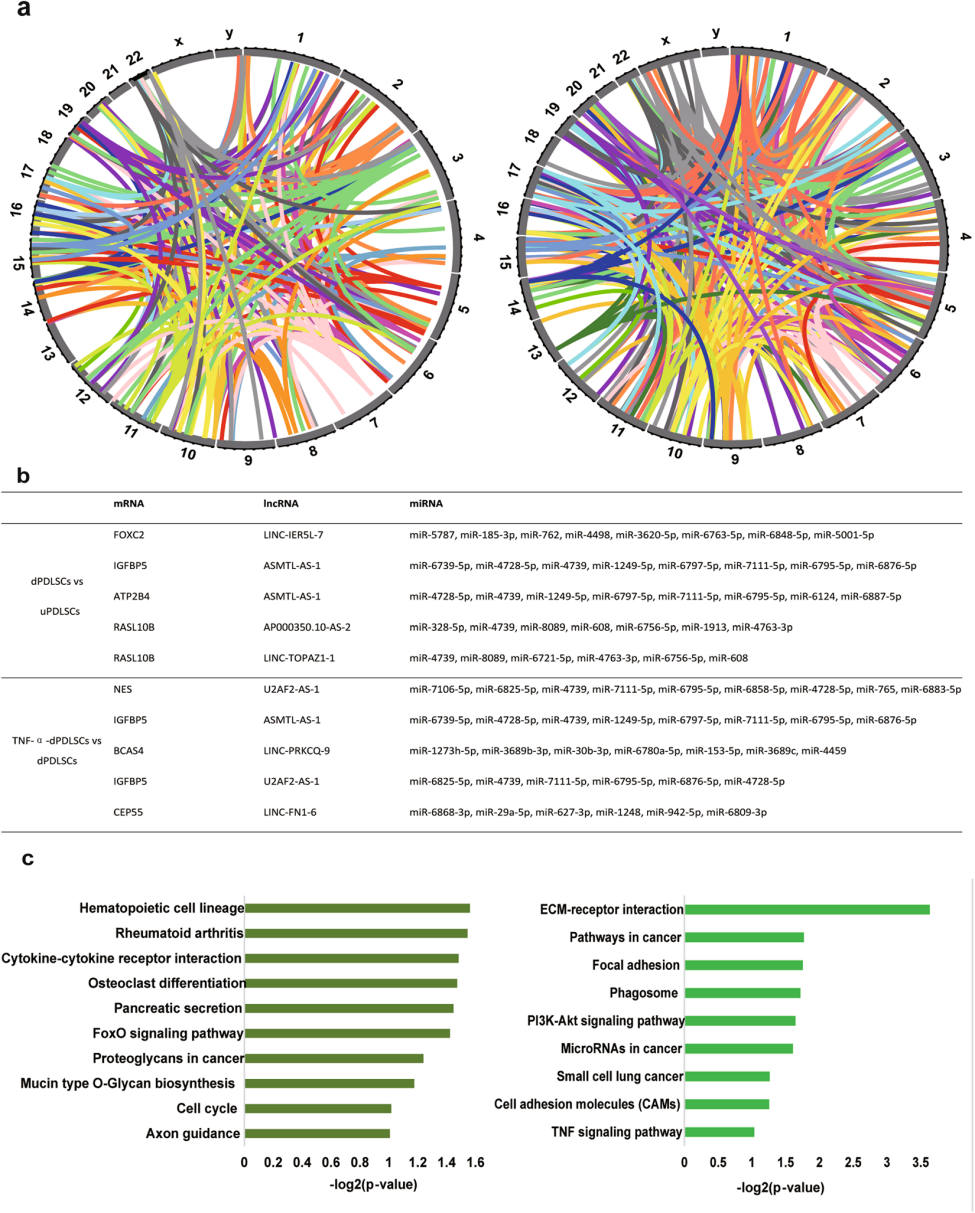
lncRNA-mRNA-related ceRNA network during osteogenic differentiation. (**a**) Circos plot showing mRNA-lncRNA ceRNA pairs for DE lncRNAs during PDLSC differentiation and TNF-α stimulation. (**b**) The top five mRNA-lncRNA ceRNA pairs and their shared miRNAs. (**c**) The significantly enriched pathways for mRNAs involved in ceRNA networks.

Recent studies indicate that lncRNAs can act as competing endogenous RNAs (ceRNAs) to indirectly regulate mRNAs through shared microRNAs. To further gain insights into the function of cell type-specific lncRNAs, we used “miRanda” to predict putative miRNA response elements (MREs) in the mRNAs and lncRNAs. In each lncRNA-mRNA ceRNA pair, we then required that the lncRNA and mRNA should share at least two miRNAs and eight unique binding sites. We calculated the Pearson correlation of each lncRNA-mRNA ceRNA pair to evaluate its miRNA-mediated strength. A Pearson correlation of 0.7 was used as the cutoff to filter the candidate lncRNA-mRNA ceRNA pairs. At last, we in total found 464 lncRNA-mRNA ceRNA interactions including 61 lncRNAs and 217 mRNAs in the DE lncRNAs between uPDLSCs and dPDLSCs, and 277 lncRNA-mRNA ceRNA interactions including 51 lncRNAs and 153 mRNAs in the DE lncRNAs between TNF-α-dPDLSCs and dPDLSCs (Fig. 5a, Supplementary Table 7). The lncRNA-mRNA ceRNA pairs were located in different chromosomes, indicating that the lncRNA-mRNA ceRNA network tends to be distantly regulated. The top 10 lncRNA-mRNA ceRNA pairs are shown in Fig. 5b. Among these lncRNA-mRNA ceRNA pairs, insulin-like growth factor binding protein 5 (*IFGBP5*) is particularly interesting. *IGFBP5* was paired with two lncRNAs, *ASMTL-AS-1* and *U2AF2-AS-1*. Of note, lncRNA *ASMTL-AS-1* was differentially expressed between both comparisons. A previous study has reported that *IGFBP5* could promote mesenchymal stem cell-mediated periodontal tissue regeneration by enhancing osteogenic differentiation and anti-inflammation potentials^27^. Our study provided new insights into the mechanism of regulating osteogenic differentiation for *IGFBP5*.

To further clarify the dysregulated ceRNA networks, we performed pathway enrichment analysis on the mRNAs in the networks using DAVID. DAVID analysis revealed that the mRNAs in the ceRNA network for DE lncRNA between uPDLSCs and dPDLSC were mainly enriched in stem cell development and differentiation-related pathways, such as the hematopoietic cell lineage and osteoclast differentiation (Fig. 5c). The mRNAs in the ceRNA network for DE lncRNA between TNF-α-dPDLSCs and dPDLSCs were mainly enriched in ECM-receptor interaction, the PI3K-Akt signaling pathway and the TNF signaling pathway (Fig. 5c). To further narrow down the important pathways, we performed IPA analysis on the mRNAs in the ceRNA networks (Supplementary Fig. 5). IPA analysis further indicated that the mRNAs in the ceRNA network for DE lncRNA between uPDLSCs and dPDLSC were mainly enriched in stem cell differentiation pathways such as ephrin receptor signaling and axonal signaling (Supplementary Fig. 5).

## Discussion

We have identified 63 novel lncRNAs in PDLSCs, and these novel lncRNAs were highly expressed in PDLSC populations. These novel lncRNAs that located near those protein-coding genes were enriched in amino acid metabolism pathways. Amino acid metabolism is known to play fundamental roles in stem cells. For instance, methionine metabolism was reported to regulate the maintenance and differentiation of human pluripotent stem cells^28^, and proline metabolism was critical for ESC maintenance and some of the earliest steps of differentiation^29,30^. The enriched amino acid metabolism pathways for novel lncRNAs in PDLSCs were leucine metabolism, isoleucine metabolism and valine metabolism, which suggests that the metabolism of these amino acids is important to osteogenic differentiation of PDLSCs. Some of the novel lncRNAs are likely involved in the metabolism of these amino acids. Further exploration of these amino acid metabolism pathways should provide insight into the specific functions of these novel lncRNAs and their roles.

lncRNAs have been reported to have tissue and linage specificity. We found that the novel lncRNAs could define the cellular identity of the three PDLSC populations according to hierarchical clustering and PCA analysis. Due to the specificity of lncRNA expression, these novel lncRNAs may not have been identified without de novo transcriptome assembly. The de novo strategy applied here could largely fill the gap in understanding the lncRNA-regulated mechanism of osteogenic differentiation.

Investigating differentially expressed lncRNAs between uPDLSCs and dPDLSCs would help to further determine the molecular mechanism of osteogenic differentiation of PDLSCs. We obtained 407 differentially expressed lncRNAs between uPDLSCs and dPDLSCs. The neighboring protein-coding genes of these DE lncRNAs were mainly enriched in serotonin receptor signaling. Previous studies have reported that serotonin receptors were necessary for the recruitment of pulpal cells involved in dental repair^26^. It was worthwhile to further clarify the function and mechanism of these DE lncRNAs involved in serotonin receptor signaling.

PDLSCs could be obtained very easily, especially in periodontitis. However, increasing evidence has shown that inflammatory microenvironments, which represents the status of the periodontitis, would inhibit the process of osteogenic differentiation of PDLSCs^9^. Therefore, it was important to understand the biological mechanism of how inflammatory microenvironments hamper differentiation, thereby improving the differentiation potential of PDLSCs derived from periodontitis in order to make better use of the stem cells. We obtained 318 differentially expressed lncRNAs between uPDLSCs and TNF-α-PDLSCs. As expected, we observed that these lncRNAs locate near protein-coding genes that are mainly enriched in inflammatory pathways. This lncRNA list is expected to be a valuable resource to study the mechanism of osteogenic differentiation of PDLSCs under inflammatory microenvironments.

LncRNAs have recently emerged as competing endogenous RNAs (ceRNAs), which play important roles in various biological processes including stem cell maintenance and differentiation. We performed a systematic investigation of expression profiling of DE lncRNAs and their correlated protein-coding genes to construct the ceRNA networks for the dysregulated lncRNAs during osteogenic differentiation of PDLSCs under inflammatory conditions. We found that protein-coding genes and lncRNAs in the ceRNA network were not a simple one-to-one but a many-to-many relationship and tended to be distantly regulated. Furthermore, we revealed that the protein-coding genes in the ceRNA network regulated by DE lncRNAs between dPDLSCs and uPDLSCs were mainly involved in pathways related to stem cell development and differentiation, such as the hematopoietic cell lineage and osteoclast differentiation. The protein-coding genes in the ceRNA network regulated by DE lncRNAs between dPDLSCs and TNF-α-PDLSCs were mainly involved in pathways related to TNF signaling and PI3K-Akt signaling. Our study provides not only new insights into the mechanism of regulating osteogenic differentiation for known regulators, such as *IGFBP5*, but also for many new regulators involved in ceRNA networks.

In summary, our study represents the first comprehensive map of lncRNA profiling underlying osteogenic differentiation of PDLSCs in the inflammatory microenvironment. Our study has identified many novel lncRNAs that were not annotated before. Moreover, we also have identified many differentially expressed lncRNAs during osteogenic differentiation. The pathway enrichment analysis indicates that these novel lncRNAs and differentially expressed lncRNAs are related to stem cell development and differentiation. In addition, our study reveals a complex lncRNA-mRNA ceRNA mechanism that may significantly contribute to the aberrant expression of critical protein-coding genes in osteogenic differentiation of PDLSCs in the inflammatory microenvironment. Our dataset should serve as a resource for the study of transcriptional regulatory networks during osteogenic differentiation of PDLSCs in the inflammatory microenvironment. We expect that further investigation of the lncRNAs identified in this study will reveal important functions of lncRNAs in the development and differentiation of PDLSCs.

## Methods

### Sample collection

The periodontal ligament (PDL) was collected from patients in the Oral and Maxillofacial Surgery Clinic of Stomatology hospital of Guangzhou Medical University. Teeth were selected from healthy patients ranging from 18 to 25 years old in whom the first or the second molar tooth was extracted for orthodontic purposes. This study was approved by the ethics committee of Stomatology Hospital of Guangzhou Medical University, and informed consent was obtained from all of the patients [#20160115]. All experiments were performed in accordance with approved guidelines and regulations from The Institute Research Medical Ethics Committee of Stomatology Hospital of Guangzhou Medical University.

### Cell culture

The teeth were soaked in media (90% DMEM, 10% FBS, 100 u/ml penicillin, 100 ug/ml streptomycin, Gibco, USA) after tooth extraction. The PDL tissue was scraped from the middle third of the root surface after washing 6–8 times with PBS (containing penicillin/streptomycin). The PDL tissue was minced into 1 mm^3^ cubes and centrifuged for 5 min in 1000 r/min. Then, the PDL tissue was digested in 3 mg/ml collagenase I (Roche, USA) and dispersed for 45 min at 37 °C. Then, an equal volume of medium was added to terminate the digestion. The solution was centrifuged at 1000 r/min for 5 min, and the supernatant was discarded. Next, the tissues were resuspended in 1 ml of medium and inoculated into 6-well plates. The cells were cultured at 37 °C in 5% CO_2_. The medium was changed every 2-3 days until the cells appeared from the edge of the tissue after 3–8 days. The cells were digested with 0.25% trypsin containing 0.1% EDTA (Roche, USA) when cell growth reached 80%. When the first-generation cells grew up to 80% confluence, the clones were isolated and cultured *in vitro* with a limiting dilution assay and expanded to 1 × 10^7^ cells in individual vessels for further cultivation. Three to four generations of poly-clonal PDLSCs were used in this study.

### RT-qPCR analysis

At 7 days after induction, total RNA was extracted according to the TRIzol Reagent (Invitrogen, USA) instructions. The RNA concentration and purity were detected using a Nano Drop ND-1000 spectrophotometer. Reverse transcription to cDNA was performed according to the rimeScriptTM RT-PCR Kit assay (Takara, Japan). The expression of osteogenic genes including *RUNX2, BGLAP* and *ALPL* was evaluated using RT-qPCR according to the SYBR Premix Ex Taq TM (Takara, Japan) instructions. GAPDH was the internal reference. Each sample obtained from three independent experiments was used for analysis of relative gene expression using the 2−ΔΔCt method.

The primers are shown below (synthesized by Generay, Shanghai, China):

***ALPL*** **:**

Forward: 5′-GCTCATGCATAACATCAGGGACA-3′

Reverse: 3′-TCGTCACTCTCATACTCCACATCAG-5′

***BGLAP*** **:**

Forward: 5′-CCCAGGGGCTACCTGTATCAA-3′

Reverse: 3′CTCCTGAAAGCCGATGTGGTC-5′

**RUNX2:**

Forward: 5′-CACTGGCGCTGCAACAAGA-3′

Reverse: 3′-CATTCCGGAGCTCAGCAGAATAA-5′

***GAPDH*** **:**

Forward: 5′-GCACCGTCAAGGCTGAGAAC-3′

Reverse: 5′-TGGTGAAGACGCCAGTGGA-3′

***GK-AS-1*** **:**

Forward: 5′-CTCAGCAAACTACCTTTCGT-3′

Reverse: 3′-GTAACCCAACCCATTGACT-5′

***LINC-PDE10A-1*** **:**

Forward: 5′-GACACACACACACACACACA-3′

Reverse: 3′- GTTCCTCCTTCCCTCCTT-5′

***SGOL1-AS-1*** **:**

Forward: 5′-TGTCGGAAAGGAGTAAGATG-3′

Reverse: 3′-ATGGAGCAAGGACCAAGT-5′

***ZNF385D-AS-1*** **:**

Forward: 5′-CCCTGCTTTATTCACCCT-3′

Reverse: 3′-GGACTTTTCCTTCCTTCATC-5′

### Western blot analysis

At 7 days post-induction, total proteins, plasmosin and nucleoproteins were extracted. The level of protein was measured by the Bio-Rad Protein Assay and BAC protein concentration assay kit (Beyotime, Shanghai, China) according to the manufacturer’s instructions. The sample was boiled for 5 min after 5-fold volume sample buffer was added and kept in storage at −80 °C. An SDS-PAGE gel was prepared based on the instructions. Transmembrane electrophoresis was performed according to the molecular weight of the proteins. Then, the PVDF membrane was incubated in blocking buffer of 5% skimmed milk. The PVDF membrane was then washed with PBS and incubated with the following primary antibodies overnight at 4 °C: anti-ALP, anti-OCN, and anti-RUNX2 (Abcam, USA). Next, membranes were incubated for 2 h at room temperature with anti-rabbit IgG secondary antibody after being placed at room temperature for 1 h. The blots were visualized using an enhanced chemiluminescence kit (Amersham Biosciences, USA) according to the manufacturer’s recommended instructions.

### Osteogenic differentiation *in vitro*

The PDLSCs were induced for osteogenic differentiation *in vitro* using osteoinductive medium (90% DMEM, 10% FBS, 100 nmol/L dexamethasone, 10 mmol/l β-glycerophosphate, 50 ug/ml vitamin C, Sigma, USA). For the TNF-α-PDLSC group, the cells were cultured with osteoinductive medium plus 10 ng/ml TNF-α (Peprotech, USA). The fourth generation of PDLSCs was digested by trypsin and resuspended. Then, the cells were inoculated into 6-well plates with a density of 2 × 10^4^ cells/cm^2^. The cells were collected at corresponding days. The detection of mineralized nodules is one of the commonly used methods to study cells’ differentiation. Alizarin red staining is the main method of staining mineralized nodules, which can make mineralized nodules dyed in deep red matter, which is due to mineralized nodules containing a lot of calcium salt, with alizarin red color reaction, resulting in a dark red compound. At 21 days after induction, we performed alizarin red staining to detect the mineralized nodules.

### RNA sequencing

At 7 days post-induction, total RNA was extracted. Polyadenylated RNA-seq was performed using standard Illumina Truseq kits, and samples were sequenced using the Illumina HiSeq 4000 PE150.

### lncRNA identification

RNA-Seq reads of each sample were aligned to the hg38 human genome using STAR (version 2.5.2b)^31^. The de novo transcriptome assembly for each sample was performed using cufflinks with the following parameters: -M = ‟rRNAmask file”,–library-type = ‟fr-firststrand,”–max-multiread-fraction = 0.25, and–3-overhang-tolerance = 2000. The transcriptome assemblies for all samples were then merged into a reconstructed transcriptome using cuffmerge with default parameters. We employed cuffcompare to compare the reconstructed transcriptome with Gencode v24 gene annotation^12^. The novel transcripts with class code “x” (exonic overlap with the reference on the opposite strand) and “u” (unknown intergenic transcript and “I” (fragment falling into an intron of the reference) were retained. Next, we filtered out the transcripts with less than two exons and with sequence lengths shorter than 200 nt. The coding capacity of all of the novel transcripts was assessed with the coding potential assessment tool (CPAT)^22^ and the predictor of long non-coding RNAs and mRNAs based on k-mer scheme (PLEK)^21^. CPAT applies logistic regression algorithm to discriminate between coding and noncoding transcripts based on the following sequence features: open reading frame size, open reading frame coverage, Fickett TESTCODE statistic and hexamer usage bias. PLEK is a computational pipeline built with an improved k-mer scheme and a support vector machine (SVM) algorithm to distinguish lncRNAs from messenger RNAs (mRNAs). The transcripts that were not predicted as protein coding by either of the two methods were retained as candidate lncRNAs. The candidate lncRNAs were merged with previous lncRNAs from Gencode^12^ and Lncipedia^23^ to build a final lncRNA reference. All novel and known lncRNAs were named according to proposed nomenclature standards^32^: LINC-[nearest protein-coding gene] for intergenic lncRNAs and [nearest protein-coding gene]-AS for antisense lncRNAs. The nearest protein-coding gene for a lncRNA was determined according to the distance from the corresponding lncRNA along the genome sequence.

### Differential expression analysis

The quantification of the expression of protein-coding genes and lncRNAs was done using RSEM^33^ with the following parameters:–paired end and–forward-prob = 0. EdgeR^34^ and DESeq2^35^ were applied to perform differential expression analysis. The protein-coding genes and lncRNAs with a p value ≤ 0.05 and an absolute value of log2foldchange ≥ 1 were treated as differentially expressed. Only the overlapped differentially expressed protein-coding genes and lncRNAs identified from DESeq2 and EdgeR were retained. Pathway enrichment analysis for differentially expressed protein-coding genes was performed using Ingenuity Pathway Analysis (IPA). For differentially expressed lncRNAs, we performed IPA on those nearest neighboring protein-coding genes to infer their functions. To build a more confident output, we have further performed LncRNAs2Pathway analysis for these differentially expressed lncRNAs.

### Construction of ceRNA network

Firstly, we extracted sequences of protein-coding genes and lncRNAs using gffread in the cufflinks toolkits. Then, the putative miRNA response elements in the protein-coding sequence and the lncRNA sequence were detected by miRanda^36^. The lncRNAs-mRNA pairs with Pearson correlation coefficient >0.7 and at least two shared miRNAs with more than 8 unique targeting sites limited in the 3′UTR region were considered as ceRNA pairs. Pathway enrichment analysis for mRNAs in the ceRNA networks was performed using IPA and DAVID^37^.

### Functional annotation of novel lncRNAs

The methods for inferring the function of novel lncRNAs are mainly based on guilt-by-association principle that states that a gene of interest with unknown functions could share functions with genes whose functions are well known in the same cluster, where links are based on common phenomenon. Genomic co-localization and co-expression are two of such common phenomenon^38–41^. To uncover the potential functions of the novel lncRNAs in PDLSCs, we performed pathway enrichment analysis using IPA on those protein-coding genes nearest to the novel lncRNAs. To further explore the potential function of novel lncRNAs, we systematically investigated co-expression patterns of lncRNAs and protein-coding genes. To do this, we computed pairwise expression correlations between genes across all samples. Expression correlations were composed of trans correlations (defined as gene pairs separated by 1 > MB distance or located on different chromosomes) and cis correlations (defined as gene pairs located within a 100-kB genomic region). Pathway enrichment analysis using IPA was performed for those protein-coding genes that were strongly positively correlated with novel lncRNAs (Pearson Correlation coefficient > 0.7).

### Availability of data and material

All raw sequencing data can be accessed from the Sequence Read Archive (project accession SRP096798).

## Electronic supplementary material

Supplementary Information

Supplementary Table 1

Supplementary Table 2

Supplementary Table 3

Supplementary Table 4

Supplementary Table 5

Supplementary Table 6

Supplementary Table 7
