# Supplementary material for: Expression and regulation of long noncoding RNAs during the osteogenic differentiation of periodontal ligament stem cells in the inflammatory microenvironment
Source: Sci Rep. 2017 Oct 25;7:13991. doi: 10.1038/s41598-017-14451-4 (PMC5656573; doi:10.1038/s41598-017-14451-4)
Supplement: Supplementary file 1 — Supplementary Information [file 41598_2017_14451_MOESM1_ESM.pdf]

Expression and regulation of long noncoding RNAs during the osteogenic differentiation of periodontal ligament stem cells in the inflammatory microenvironment

Qingbin Zhang[1#], Li Chen[2,3#], Shiman Cui[1#], Yan Li[2,3#], Qi Zhao[2], Wei Cao[1], Shixiang Lai[1], Sanjun Yin[4], Zhixiang Zuo[2*], Jian Ren[2,3*]

[1]Key Laboratory of Oral Medicine, Guangzhou Institute of Oral Disease, Stomatology Hospital of Guangzhou Medical University, Guangzhou 510140, China

[2]Sun Yat-sen University Cancer Center, State Key Laboratory of Oncology in South China, Collaborative Innovation Center for Cancer Medicine, Sun Yat-sen University, Guangzhou 510060, China

[3]State Key Laboratory of Biocontrol, School of Life Sciences, Sun Yat-sen University, Guangzhou 510275, China

[4]Total Genomics Solution (TGS) Institute, Shenzhen 518000, China

# Contributed equally

*Correspondence to: Tel/Fax: +86 20 39943788; Email: renjian.sysu@gmail.com (Jian Ren), zuozhx@sysucc.org.cn (Zhixiang Zuo)

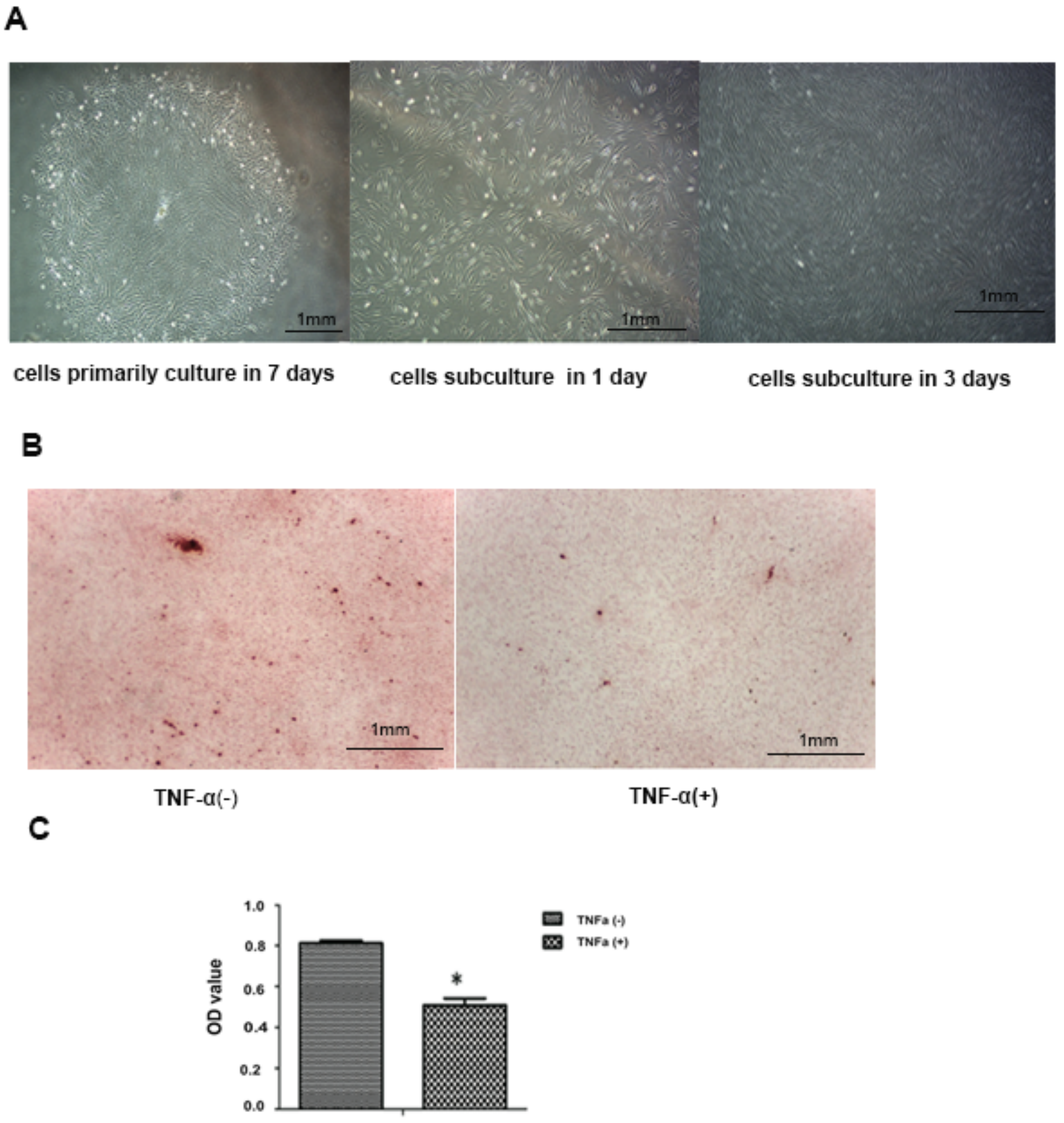

Supplementary Fig. 1: Clone-like appearance of PDLSCs. (A) From left to right: cells primarily cultured for 7 days, cells subcultured for 1 day and cells subcultured for 3 days. (B) Alizarin red staining of dPDLSCs (left) and TNF-α-PDLSCs (right). (C) Quantitative analysis of Alizarin red staining.

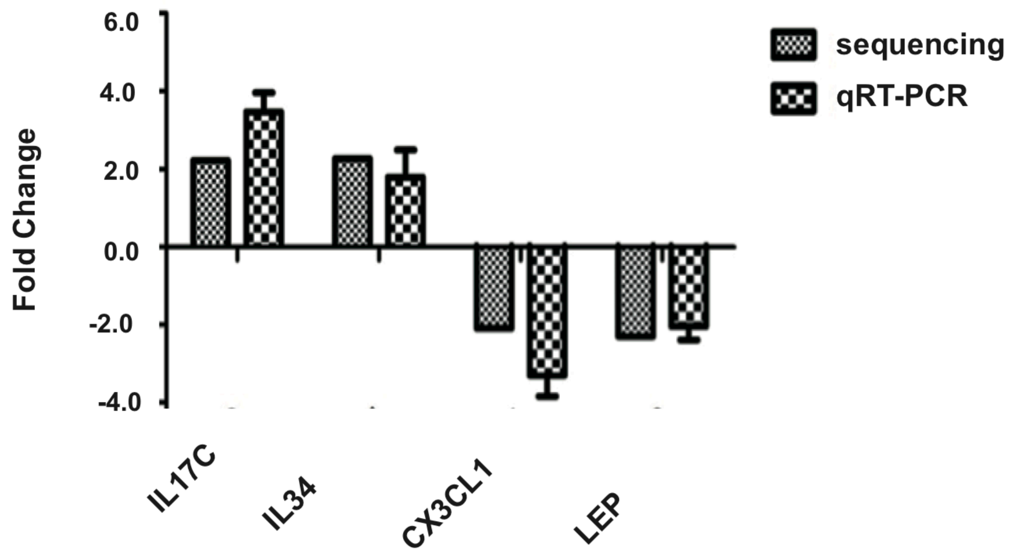

Supplementary Fig. 2: The expression change of four protein-coding genes validated by RT-PCR.

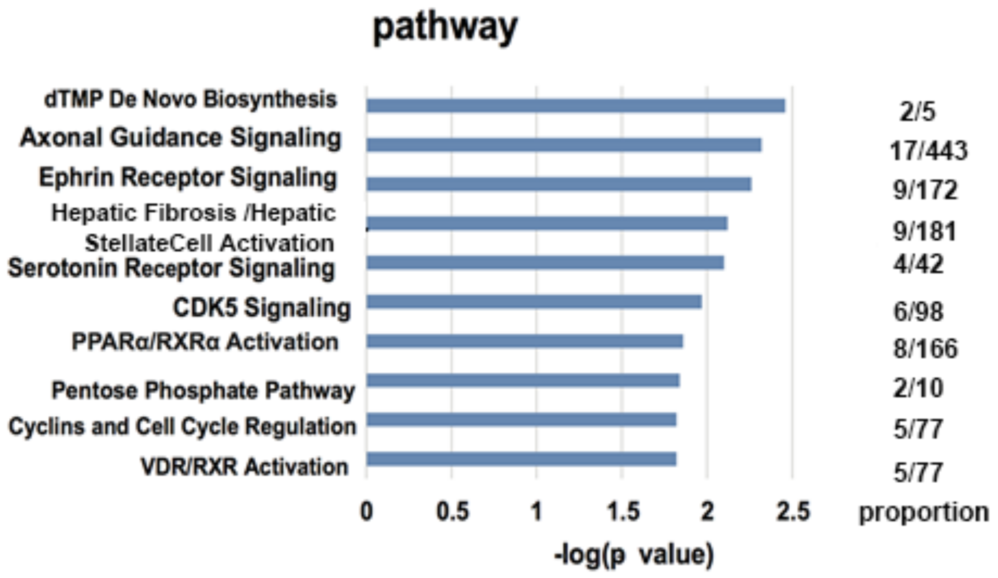

Supplementary Fig. 3: Significantly enriched pathways for neighboring protein-coding genes for DE lncRNAs between uPDLSCs and dPDLSCs.

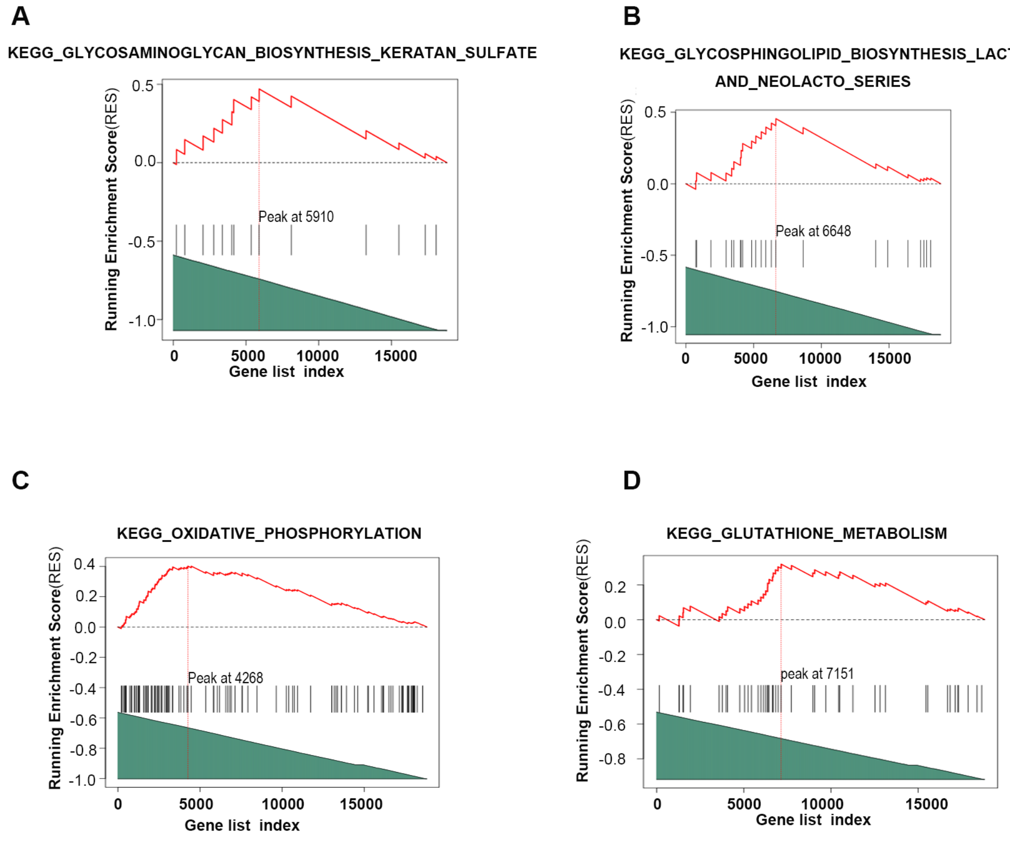

Supplementary Fig. 4: (A) (B)The top two significantly enriched pathways for DE lncRNAs between uPDLSCs and dPDLSCs via LncRNAs2Pathways. (C) (D) The top two significantly enriched pathways for DE lncRNAs between dPDLSCs and TNF-α-dPDLSCs via LncRNAs2Pathways.

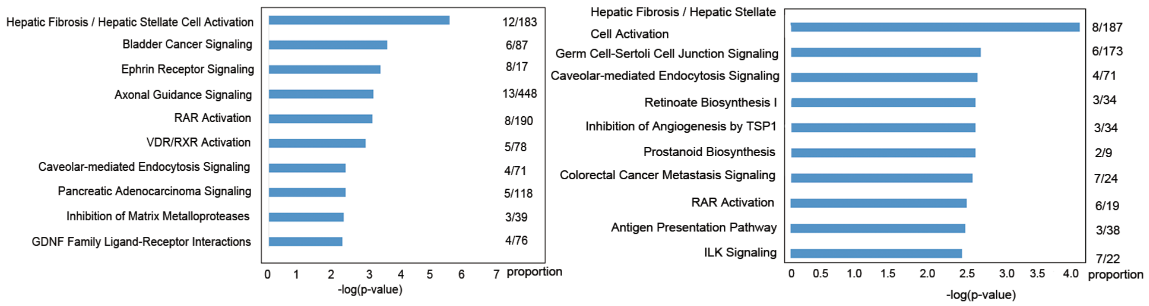

Supplementary Fig. 5: The significantly enriched pathways for mRNAs involved in ceRNA networks via IPA.

Supplementary Table 1: The final lncRNA reference consisted of 31,822 previously identified lncRNAs and 63 novel lncRNAs not annotated in lncRNA databases.

Supplementary Table 2: Differentially expressed lncRNAs identified by DESeq2 and EdgeR in dPDLSCs compared to uPDLSCs.

Supplementary Table 3: Differentially expressed lncRNAs identified by DESeq2 and EdgeR in TNF-α-dPDLSCs compared to dPDLSCs.

Supplementary Table 4: Differentially expressed protein-coding genes identified by DESeq2 and EdgeR in dPDLSCs compared to uPDLSCs.

Supplementary Table 5: Differentially expressed protein-coding genes identified by DESeq2 and EdgeR in TNF-α-dPDLSCs compared to dPDLSCs.

Supplementary Table 6: A. Significantly enriched pathways for DE lncRNAs between uPDLSCs and dPDLSCs via LncRNAs2Pathways analysis. B. Significantly enriched pathways for DE lncRNAs between dPDLSCs and TNF-α-dPDLSCs via LncRNAs2Pathways analysis.

Supplementary Table 7: A. the lncRNAs, mRNAs and shared miRNAs of lncRNA-mRNA ceRNA interactions in the DE lncRNAs between dPDLSCs and uPDLSCs. B. the lncRNAs, mRNAs and shared miRNAs of lncRNA-mRNA ceRNA interactions in the DE lncRNAs between TNF-α-dPDLSCs and dPDLSCs.
